# IL-28B Genetic Variants Determine the Extent of Monocyte-Induced Activation of NK Cells in Hepatitis C

**DOI:** 10.1371/journal.pone.0162068

**Published:** 2016-09-01

**Authors:** Benjamin Krämer, Claudia Finnemann, Beatriz Sastre, Philipp Lutz, Andreas Glässner, Franziska Wolter, Felix Goeser, Pavlos Kokordelis, Dominik Kaczmarek, Hans-Dieter Nischalke, Christian P. Strassburg, Ulrich Spengler, Jacob Nattermann

**Affiliations:** 1 Department of Internal Medicine I, University of Bonn, Bonn, Germany; 2 German Center for Infection Research (DZIF), Bonn, Germany; 3 Department of Infectious Diseases, Institute for Health Research (IRYCIS), University Hospital Ramón y Cajal, Madrid, Spain; 4 AIDS Research Network (RIS-RETICS), Madrid, Spain; University of Sydney, AUSTRALIA

## Abstract

**Background:**

Immuno-genetic studies suggest a functional link between NK cells and λ-IFNs. We recently showed that NK cells are negative for the IFN-λ receptor IFN-λR1 and do not respond to IFN-λ, suggesting a rather indirect association between *IL-28B* genotype and NK cell activity.

**Methods:**

A total of 75 HCV(+) patients and 67 healthy controls were enrolled into this study. *IL-28B* (rs12979860) and *IFNL-4* (rs368234815) genotypes were determined by rtPCR. Total PBMC, monocytes, and NK cells were stimulated with IL-29, the TLR-7/8 agonist R848, or a combination of both. NK cell IFN-γ response was analysed by FACS. IL-12 and IL-18 secretion of monocytes was studied by ELISA. In blocking experiments anti-IL-12/anti-IL-18 were used.

**Results:**

Following stimulation of total PBMCs with R848 we found NK cell IFN- γ responses to vary with the *IL-28B* genotype, with carriers of a T/T genotype displaying the lowest frequency of IFN-γ(+)NK cells. When isolated NK cells were studied no such associations were observed, indicating an indirect association between *IL-28B* genotype and NK cell activity. Accordingly, we found R848-stimulated monocytes of patients with a T/T genotype to be significantly less effective in triggering NK cell IFN- γ production than monocytes from carriers of a non-T/T genotype. In line with these findings we observed monocytes from T/T patients to secrete significantly lower concentrations of IL-12 than monocytes from non-T/T individuals.

**Conclusions:**

Our data indicate that monocytes from carriers of an *IL-28B* T/T genotype display a reduced ability to stimulate NK cell activity and, thus, provide a link between *IL-28B* genotype and NK functions.

## Introduction

Infection with the hepatitis C virus (HCV) is a major cause of blood-borne hepatitis worldwide. The majority of patients exposed to HCV develop chronic infection which is associated with a significant risk to develop chronic liver disease, including cirrhosis and hepatocellular carcinoma.

Host genetic factors are considered to importantly modulate the immune response against invading pathogens. Accordingly, numerous genetic variants have been proposed to be associated with spontaneous and/or treatment-induced clearance of HCV infection. However, only few of these findings could unequivocally be confirmed in independent studies [1,2]. In three genome wide association studies a single nucleotide polymorphism (SNP) in close proximity to the *interleukin 28 B* (*IL-28B*) gene, which encodes for the type III interferon lambda 3 (IFN-λ3), has been shown to be significantly associated with response to IFN-based therapy of chronic hepatitis C and with the natural course of acute hepatitis C [3–5]. More recently, Prokunina-Olsson et al. identified a genetic variant rs368234815 (TT or ΔG) upstream of the *IFNL3* gene, which creates (ΔG) or disrupts (TT) an open reading frame in a new gene, designated IFNL4 [6]. This polymorphism is in high linkage disequilibrium with rs12979860 and was found to be more strongly associated with HCV clearance than the *IL28B* rs12979860 variant in individuals of African ancestry, but to provide comparable information in Europeans and Asians. Moreover, Bibert and co-workers showed that in PBMCs induction of *IL28B* and *IP-10* mRNA was dependent on the TT/-G variant, but not rs12979860 [7]. However, the mechanism by which this *IFNL4* variant is associated with spontaneous and/or treatment-induced clearance or HCV infection are still incompletely understood.

In addition, variants in the killer cell immunoglobulin-like receptors (KIR) gene locus, encoding a highly polymorphic family of natural killer cell receptors, have repeatedly confirmed to be associated with response to therapy and outcomes of hepatitis C [8–10]. In functional studies, Ahlenstiel and co-workers demonstrated that this association might be attributed to a differential natural killer (NK) cell activation and function in the context of this KIR/HLA interaction [11].

Interestingly, two independent studies demonstrated that the *IL-28B* genotype in combination with specific variants in KIR/HLA gene loci synergistically affect outcome of hepatitis C [12,13]. Thus, it was suggested that NK cell functions are influenced by IFN- λ [12–14]. However, human NK cells have been shown to be negative for IFN-λR1, the natural receptor for λ-IFNs, and, thus, a direct modulation of NK cell activity by λ-IFNs seems unlikely. Accordingly, we and others recently showed that human NK cells do not respond to stimulation with λ-IFNs [15,16], suggesting a rather indirect association between *IL-28B* genotype and NK cell functions.

## Materials and Methods

### Study cohort

A total of 75 Caucasian patients with chronic hepatitis C, all from the Bonn area in Germany, were enrolled into this study. All patients were negative for HIV or HBV co-infection and none of the patients received antiviral treatment at the time of blood donation. As a control, blood of 67 healthy donors were obtained from the blood transfusion service of the university hospital Bonn. Detailed patients´ characteristics are given on Table 1.

**Table 1:** Patient characteristics.

HCV (+) patients			
	IL28B SNP (rs1279860)		
	C/C	C/T	T/T
**Number**	28	30	17
Sex, female ^a^^)^	10(36%)	9(30%)	7(41%)
Age ^b^^)^	55 (14)	58 (14)	52(11)
**HCV status**			
HCV load (IU/ml) ^b^^)^	2,671e+006(2,747e+006)	1,443e+006(1,799e+006)	1,165e+006(1,394e+006)
Genotype 1	24(85%)	23(77%)	16(94%)
Genotype 2	4(15%)	5(17%)	0(0%)
Genotype 4	0(0%)	2(6%)	3(17%)
**Liver status**			
**ALT** ^b^^)^[U/l]	91,46(86,9)	77,9(67,14)	104,2(65,7)
**AST** ^b^^)^[U/l]	64,43(46,23)	64,52(58,44)	82,06(54,24)
**bilirubin** ^b^^)^[mg/dl]	0,72(0,29)	0,54(0,23)	0,97(1,3)
**gGT** ^b^^)^[U/l]	76,9(70,6)	157,1(147,4)	139,3(118,4)
**AP** ^b^^)^[U/l]	82,6(30,8)	88,6(27,1)	104,1(45,27)
**IFNL-4 SNP (rs368234815)**			
**TT/TT**	28 (100%	0(0%)	0(0%)
**TT/ΔG**	0(0%)	30 (100%)	0(0%)
**ΔG/ΔG**	0(0%)	0(0%)	17(100%)

a) number of cases (number/total in %)

b) mean (Std. Dev)

The study was approved by the Ethics Commission for the Medical Faculty of the University Bonn and all participants provided informed written consent.

### DNA Extraction and Genotyping

Genomic DNA was extracted from 50 μL EDTA-blood using the QIAamp Blood Mini Kit (Qiagen, Hilden, Germany) according to the manufacturer's protocol. Determination of the *IL-28B* (rs12979860) and *IFNL4* (rs368234815) genotype was performed by LightCycler real time PCR (Roche, Mannheim, Germany) using a commercial LightSNiP (SimpleProbe) assay purchased from TIB-MolBiol (Berlin, Germany) according to the manufacturer's recommendations.

### HuH7_HCV_Replicon cells

HuH7_HCV_Replicon cells [17] were kindly provided by Lohmann and Bartenschlager (Department of Molecular Virology, University of Heidelberg, Germany). Cells were grown in high glucose (4.5g/l) DMEM supplemented with glutamine (PAA, Germany), 10% FCS, nonessential amino acids (Biochrom, Germany), and 1% penicillin/streptomycin (Sigma-Aldrich, USA). Blasticidin S hydrochloride (3μg/ml) and G418 (1mg/ml) (both PAA, Germany) were added to cells to maintain subgenomic replicons. HuH7_HCV_Replicon cells were splitted twice a week and were seeded at a dilution of 1:4.

### Primary human hepatic stellate cells

Primary activated human hepatic stellate cells (HSC) obtained from ScienCell (San Diego, CA, USA) have been well characterized [18]. HSC were cultured for 2–4 passages in defined Stellate Cell Medium (SteCM, ScienCell) supplemented with 2% fetal bovine serum, 5 ml stellate cell growth supplement, 10 U/ml penicillin and 10 μg/ml streptomycin (all ingredients obtained from ScienCell) at 37°C with 5% CO_2._ Then, cells were cryopreserved until further use.

Two days before HSC were used in an experiment, the cells were thawed and cultured in SteCM medium. Then cells were harvested, washed, checked for viability using trypan blue, and used in the respective experiments.

### PBMC stimulation

Peripheral blood mononuclear cells (PBMC) were isolated using Ficoll-Paque gradient centrifugation (Biochrom AG, Berlin, Germany) and directly cryopreserved until further usage. Frozen PBMCs were thawed in RPMI 1640 medium (Gibco, USA) supplemented with HS-Nuclease (25U/ml, MoBiTec, Göttingen, Germany) for 10 min, washed twice with PBS and cultured for 16h in the presence of TLR-7/8 agonists R848 (200ng/ml, Enzo Life Sciences, Lörrach, Germany) combined with or without 100ng/ml recombinant IL-29 (Immunotools, Germany) in complete RPMI 1640 medium (10% FCS/ 1% pen/strep). Pre-activated PBMC were then co-cultured with HuH7_HCV_Replicon cells (alternatively with primary HSC) at an effector:target (E:T) ratio of 1:1 at 37°C for 5h.

### Monocyte/NK cell co-cultures

Monocytes and NK cells were isolated from total PBMCs by negative selection using Pan Monocyte Isolation Kit and NK Cell Isolation Kit, respectively (Miltenyi, Bergisch Gladbach, Germany). The purity of isolated cells was routinely assessed by flow cytometry, and preparations with >90% purity were used for experiments. Monocytes and NK cells were co-cultured at a ratio of 3:1 in complete RPMI 1640 medium. Following 16h of pre-stimulation with R848 (200ng/ml) or Poly I/C (100ng/ml; Sigma, USA), respectively, in the presence or absence of recombinant IL-29 (100ng/ml) cells were co-incubated with HuH7_HCV_Replicon cells at an E:T ratio of 1:1 at 37°C for 5h.

In blocking experiments co-cultures were performed in the presence of mAbs specific for IL-12-p40 (10μg/ml, Biolegend, USA) and/or IL-18 (10μg/ml, Biolegend, USA).

### Intracellular IFN-γ production

After co-cultures in the presence of brefeldin A (5μg/ml; Biolegend, USA), cells were harvested and washed using PBS. Then, cells were stained with anti-CD56, anti-CD16, anti-CD3 mAbs (all from Biolegend, USA), fixed and permeabilized using Cytofix/Cytoperm (BD Biosciences, Germany), followed by intracellular staining with anti-IFN-γ mAb (Biotechne, Minneapolis, USA). Before antibody staining, cells were treated with Zombie Aqua Fixable Viability Kit (Biolegend, USA) to exclude dead cells from analysis. Cell analysis was performed on a FACSCanto II flow cytometer (BD Biosciences, Germany) using the Flowjo 10.1 software package (Treestar, USA).

### CD107a degranulation assay

Cytotoxic activity of NK cells was assessed in a CD107a degranulation assay as described before [15]. In brief, pre-activated PBMC were co-cultured with primary HSC in the presence of anti-CD107a mAb for 1h (BD, Heidelberg, Germany). Then, GolgiStop (1:100; BD, Heidelberg, Germany) was added and cells were co-cultured for an additional 4h. For detection of surface proteins cells were stained with anti-CD56, anti-CD16, anti-CD3 mAbs (all from Biolegend, USA) and fixed using Cellfix solution (BD Biosciences, Germany). Viability staining was performed by Zombie Aqua Fixable Viability Kit. Cell analysis was performed by flow cytometry.

### TLR-7/8 expression

Purified monocytes were incubated with or without 200 ng/ml R848 for 16h. Then, cells were fixed and permeabilized using Cytofix/Cytoperm (BD Biosciences, Germany), followed by intracellular staining with anti-TLR-7 (ebioscience, USA) /anti-TLR-8 (BD, Germany) mAbs and flow cytometric analysis. Expression of TLR7 and TLR8, respectively, was determined as geometric MFI (geometric mean fluorescence intensity). Viability staining was performed by Zombie Aqua Fixable Viability Kit.

### STAT-1/5 phosphorylation

Purified monocytes were stimulated with 200 ng/ml R848 for 90 min. Stimulated cells were immediately fixed with 4% paraformaldehyde for 10 min, washed twice with PBS, incubated with ice-cold methanol for 10 min at 4°C and stored in -20°C freezer. After at least one day in -20°C storage cells were thawed in complete RPMI 1640 medium, washed twice with PBS, followed by staining with anti-pSTAT1 (eBioscience,USA) /anti-pSTAT5 (BD Biosciences, Germany) mAbs in 5% BSA-PBS and flow cytometric analysis. Expression was determined as MFI by using geometric mean values.

### Cytokine release of monocytes

200,000 purified monocytes were seeded in 48-well plates and stimulated with R848 (200ng/ml) in complete RPMI 1640 medium for 16h. Cell suspension was transferred into a centrifuge tube and centrifuge at 1,500 rpm for 10 min at 4°C. Concentrations of IL-12p40, IL-12-p70 and IL-18 in culture supernatants was determined by ELISA (ebioscience, USA).

### Statistics

Statistical analyses were performed using GraphPad Prism software (version 6; GraphPad Software, Inc., San Diego, CA) and the SPSS 22.0 (SPSS, Inc., Chicago, IL) statistical package. One-Way ANOVA was used to compare NK cell cytokine production, monocyte cytokine level and monocyte protein expression between different *IL-28B* genotypes. Repeated measures ANOVA were used to compare NK cell cytokine production between unstimulated and more than one different stimulation conditions. Wilcoxon matched paired tests were used to compare NK cell cytokine production, monocyte cytokine levels and protein expression between unstimulated and stimulated samples. A two-sided P value <0.05 was considered significant (* P <0.05, ** P <0.01, *** P <0.001, n.s. “not significant”).

## Results

### Human NK cells are insensitive to stimulation with IL-29

**Fig 1:**
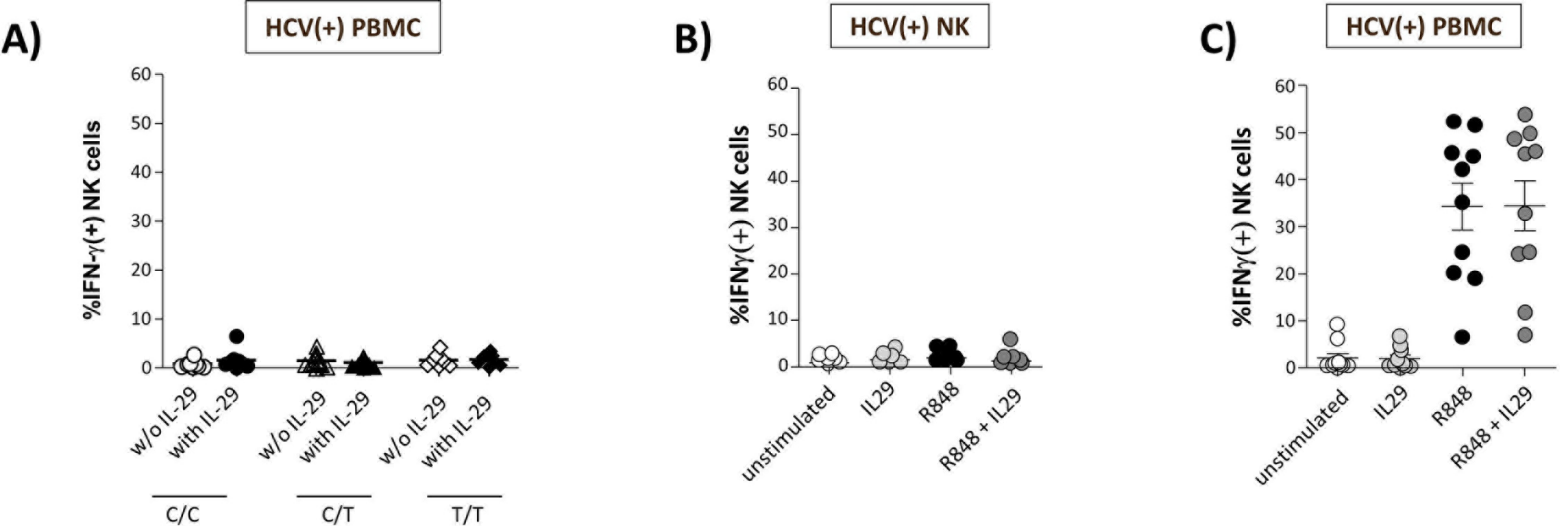
Human NK cells are insensitive to stimulation with IL-29. Purified NK cells from HCV(+) patients carrying an *IL-28B* C/C (n = 8), C/T (n = 12), or T/T (n = 7) genotype where cultured in the presence or absence of IL-29 (100ng/ml) overnight, then co-cultured with HuH7_HCV_Replicon cells for 5h and tested for IFN- γ production **(A**). Total PBMC (n = 27) **(B)** from HCV(+) patients were stimulated with IL-29 (100ng/ml), R848 (200ng/ml), or a combination of both overnight. Then, PBMC were co-cultured with HuH7_HCV_Replicon cells for 5h and tested for IFN-γ production.

We previously demonstrated NK cells to lack expression of the IFN- λR1 receptor. Accordingly, purified NK cells were found to be insensitive towards stimulation with λ-IFNs [15]. Thus, we tested whether λ-IFNs might activate a peripheral blood cell type other than NK cells which then in turn stimulates NK cell activity. To this end, total PBMC from HCV(+) patients were stimulated with IL-29 and then IFN- γ production of NK cells was studied following co-incubation with HuH7_HCV_Replicon cells. However, we did not observe any substantial increase in NK cell activity following stimulation with IL-29, irrespective of the *IL-28B* genotype (Fig 1A).

Next, we tested whether a co-stimulatory signal might be required for lL-29 induced NK cells activation. For this purpose, PBMC from HCV(+) patients were stimulated with the TLR-7/8 ligand R848 in the presence or absence of IL-29 following co-incubation with HuH7_HCV_Replicon cells. As is shown in Fig 1B, frequency of IFN-γ(+) NK cells significantly increased following R848 stimulation of total PBMCs. However, this effect was found to be independent of IL-29 co-stimulation.

### *IL-28B* genotype-dependent activation of NK cells

**Fig 2:**
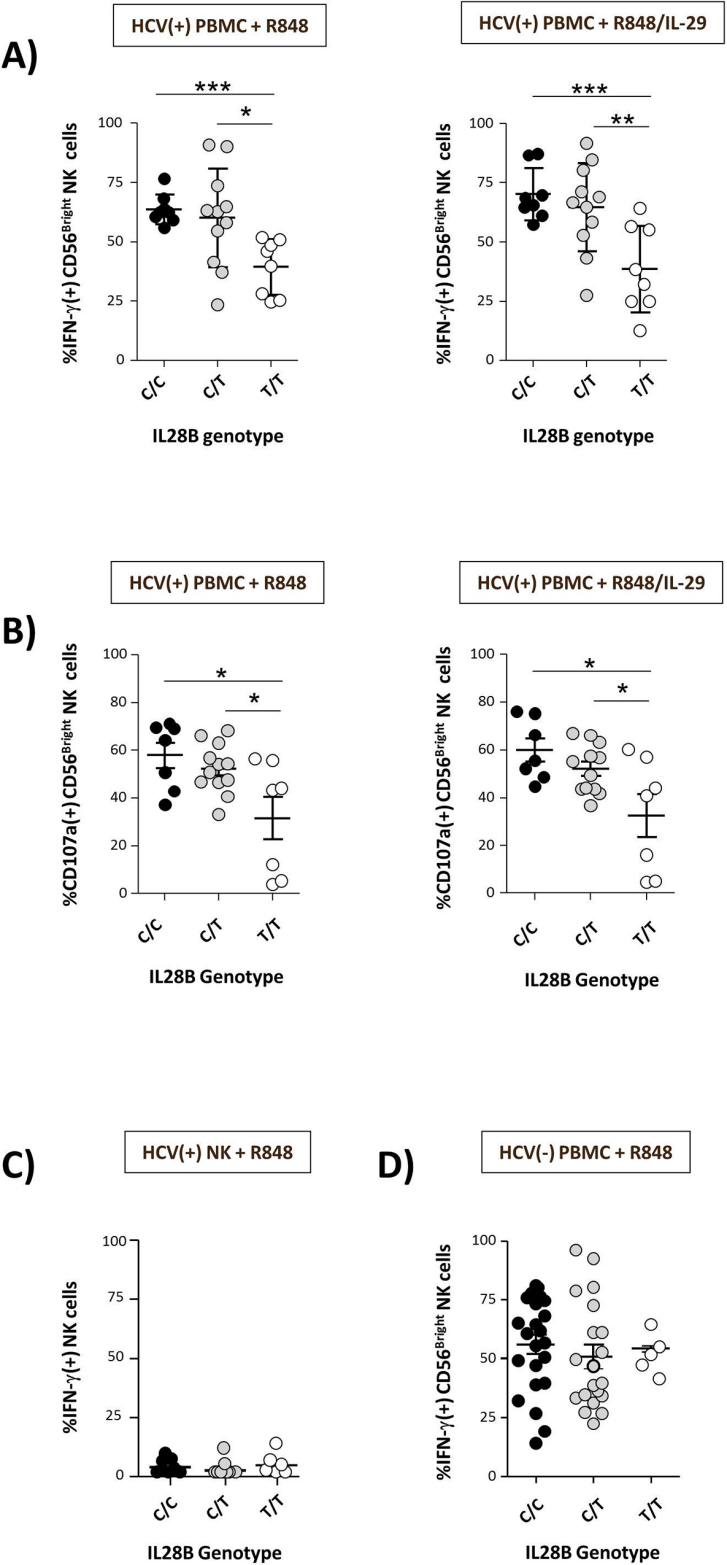
R848-mediated activation of NK cells is *IL-28B* genotype-dependent. Total PBMCs from HCV patients with different *IL-28B* genotypes were pre-stimulated with either R848 or R848 + IL-29 and then co-cultured with HuH7_HCV_Replicon cells or hepatic stellate cells, respectively. After 5h of co-incubation IFN-γ production (against HuH7_HCV_Replicon cells; C/C, n = 8; C/T, n = 12; T/T, n = 7) **(A)** or CD107a expression (against hepatic stellate cells; C/C, n = 7; C/T, n = 12; T/T, n = 7) **(B)** of CD56^Bright^ NK cells was studied by FACS analysis. As a control IFN-γ production of purified HCV(+) NK cells (C/C, n = 9; C/T, n = 10; T/T, n = 6) pre-stimulated with R848 was analysed following co-incubation with HUH7_HCV_replicon **(C)**. In addition, PBMCs from healthy donors (C/C, n = 23; C/T, n = 20; T/T, n = 5) were pre-stimulated with R848, co-incubated with HuH7_HCV_Replicon cells and then tested with respect to IFN-γ production of CD56^Bright^ NK cells **(D)**.

We next analysed whether R848-mediated stimulation of NK cells might differ between carriers of different *IL-28B* genotypes. Indeed, we observed R848-induced activation of NK cells co-incubated with HuH7_HCV_Replicon cells to be associated with the *IL-28B* polymorphism with carriers of a T/T genotype displaying significantly lower NK cell IFN-γ production than carriers of a non-T/T genotype (Fig 2A, left graph; S1 Fig, exemplary flow cytometric gating strategy). Of note, this effect was also found in the absence of IL-29 co-stimulation (Fig 2A, right graph) and was found to be independent of viral load or transaminase levels (S4 Fig). Similar observations were made regarding R848-induced NK cell degranulation following co-incubation with hepatic stellate cells. (Fig 2B). No such association was observed when isolated NK cells were studied, indicating an indirect effect (Fig 2C). Moreover, an association between NK cell activity and the *IL-28B* genotype could only be confirmed when PBMC from HCV patients were studied but not when cells obtained from healthy controls were analysed (Fig 2D).

### Monocyte-induced NK cell activation is associated with the *IL-28B* genotype

**Fig 3:**
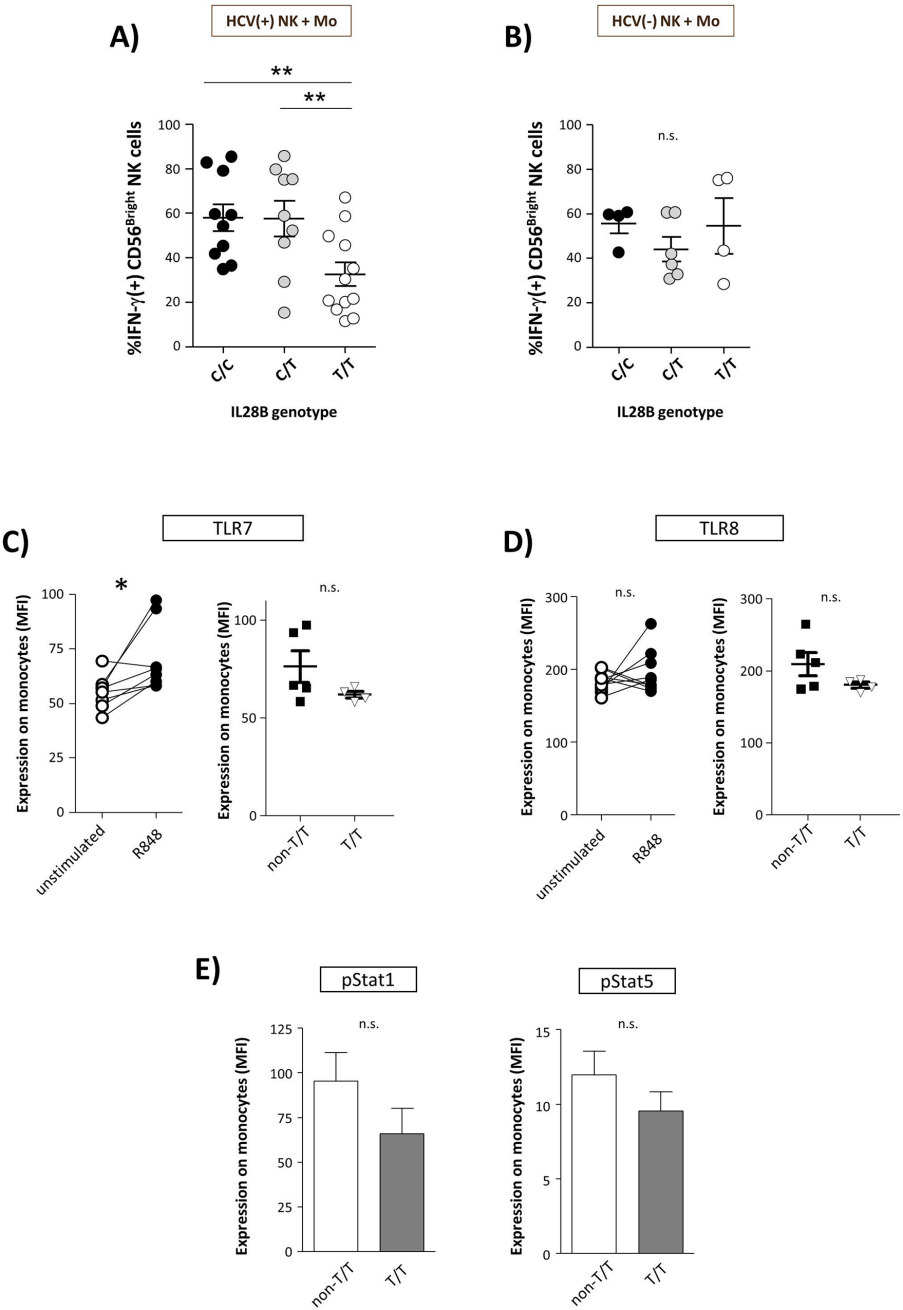
Monocyte-induced NK cell activation is associated with the *IL-28B* genotype. NK cells from HCV(+) patients (C/C, n = 10; C/T, n = 9; T/T, n = 12) **(A)** and healthy donors (C/C, n = 4; C/T, n = 6; T/T, n = 4) **(B)** were co-cultured with autologous monocytes in the presence of R848 and then tested for IFN-γ production of CD56^Bright^ NK cells following co-incubation with HuH7_HCV_Replicon cells. Purified HCV(+) monocytes were stimulated with R848 and then analysed with respect to expression (MFI) of TLR-7(non-T/T, n = 5; T/T, n = 4) **(C)**, TLR-8 (non-T/T, n = 5; T/T, n = 4) **(D),** and phosphorylated Stat-1 and Stat-5 (non-T/T, n = 5; T/T, n = 4) **(E)**, respectively.

As monocytes have repeatedly been shown to stimulate NK cell functions we next tested whether NK cell/monocyte interactions might differ among carriers of a specific *IL-28B* genotypes. To this end, NK cells were co-cultured with autologous monocytes in the presence of R848 and then tested against HuH7_HCV_Replicon cells. As is shown in Fig 3A, we found monocytes from carriers of an *IL-28B* T/T genotype to be significantly less effective in triggering NK cell IFN-γ production than monocytes obtained from patients with a non-T/T genotype. Again, this effect was seen only in patients with chronic hepatitis C but not in healthy controls (Fig 3B). In order to analyse this phenomenon in more detail we next performed cross-culture experiments. When monocytes from HCV infected patients were used to activate NK cells from healthy subjects we observed an *IL-28B* genotype dependent induction of NK cell IFN-γ production, whereas no such effects could be observed when monocytes from healthy subjects were co-cultured with NK cells from HCV infected patients (S3 Fig).

Then, we studied whether *IL-28B* genotype dependent activation of NK cells by monocytes was specific for the TLR7/8 pathway. To this end, monocytes were stimulated with the TLR3 agonist Poly-I/C and then tested for triggering NK cell IFN- γ production [19]. In these experiments Poly-I/C stimulated monocytes from HCV infected patients with a C/C genotype were found to be more effective in inducing NK cell IFN-γ production than monocytes from HCV patients with an *IL-28B* non-C/C genotype, indicating a more general effect (S2 Fig).

Next, we analysed whether monocyte expression of the R848 receptors TLR-7/TLR-8 was associated with the *IL-28B* genotype. R848 significantly increased TLR-7 expression (Fig 3C, left graph), and this effect was slightly stronger in carriers of a non-T/T genotype than in *IL-28B* T/T patients (Fig 3C, right graph). However, this difference was not statistically significant. Similar observations were made with respect to expression of TLR-8 (Fig 3D).

Finally, we studied monocyte expression of pSTAT1 and pSTAT5, which are involved in the TLR-7/8 signalling pathway [20,21]. However, expression of these molecules did not differ significantly between monocytes from patients carrying a T/T or a non-T/T *IL-28B* genotype (Fig 3E).

### Monocyte production of IL-12 is associated with the *IL-28B* genotype

Monocyte-induced activation of NK cells has been shown to be dependent on IL-12 and/or IL-18 [22]. Therefore, we next studied the potential association between the *IL-28B* genotype and monocyte production of IL-12 and IL-18, respectively, in hepatitis C.

**Fig 4:**
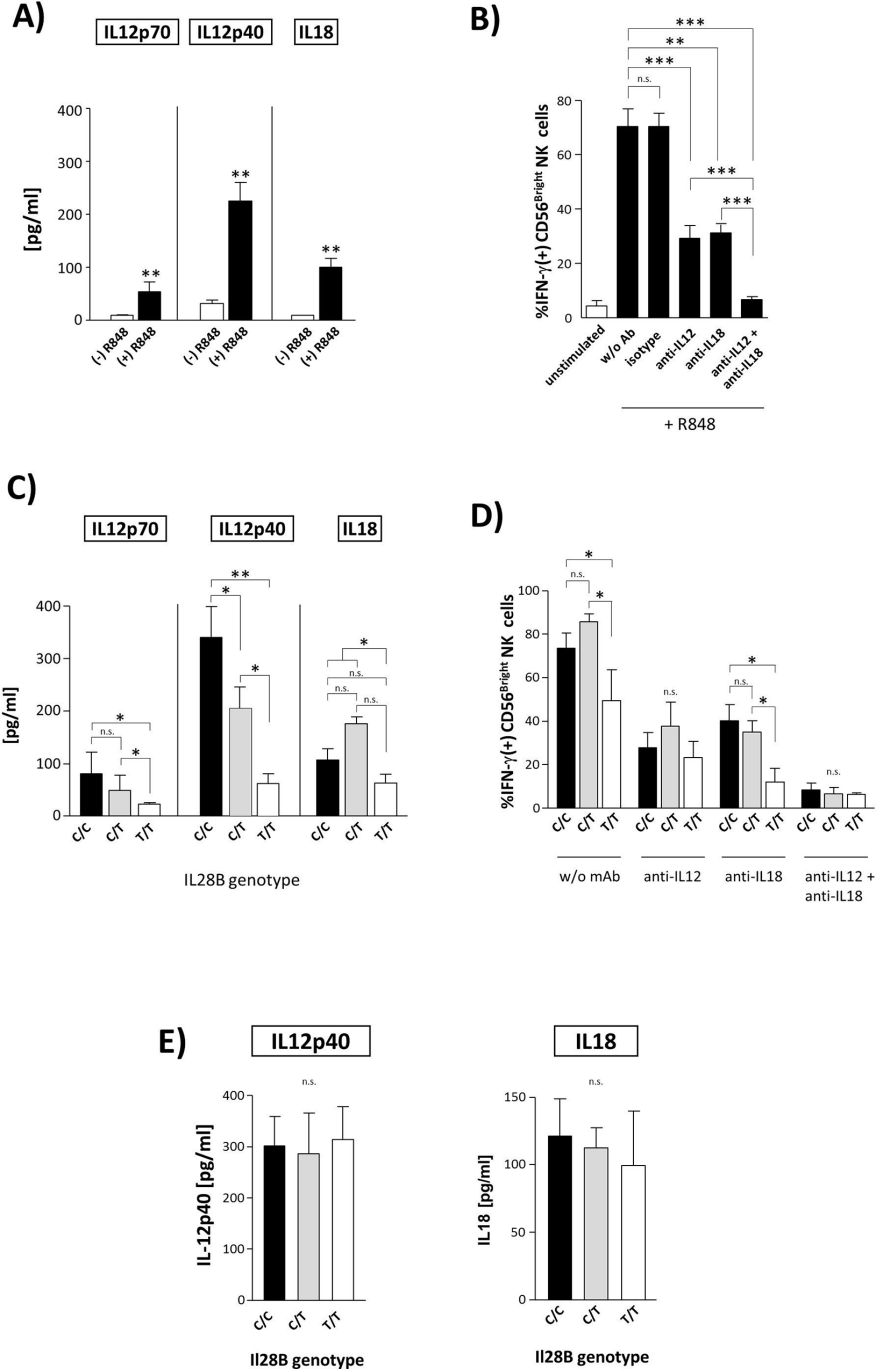
Monocyte production of IL-12 is associated with the *IL-28B* genotype. Purified monocytes from HCV(+) patients were stimulated with R848 and then tested for expression of IL-12-p70 (n = 12), IL-12-p40 (n = 19), and IL-18 (n = 15), respectively **(A).** NK cells from HCV(+) patients (n = 16) were co-cultured with R848 stimulated autologous monocytes and HuH7_HCV_Replicon cells in the presence or absence of anti-IL-12, anti-IL-18, or a combination of both mAbs. Then, IFN-γ production of CD56^Bright^ NK cells was studied **(B)**. Purified monocytes from HCV(+) patients were stimulated with R848. After 16h the resulting supernatants were harvested and tested for concentrations of IL-12-p40 (C/C, n = 7; C/T, n = 8; T/T, n = 4), IL-12-p70 (C/C, n = 5; C/T, n = 3; T/T, n = 4), and IL-18 (C/C, n = 7; C/T, n = 4; T/T, n = 4), respectively, by ELISA **(C)**. Next, R848-stimulated monocytes from HCV(+) patients that were stratified according to the *IL-28B* genotype (C/C, n = 7; C/T, n = 3; T/T, n = 6) were cultured with NK cells. Then, IFN-γ production of CD56^Bright^ NK cells was tested following co-incubation with HuH7_HCV_replicon in the presence of anti-IL-12 and/or anti-IL-18 **(D)**. As a further control, purified monocytes from healthy donors were stimulated with R848. Then, concentrations of IL-12-p40 (C/C, n = 6; C/T, n = 5; T/T, n = 3; left graph) and IL-18 (C/C, n = 6; C/T, n = 7; T/T, n = 4; right graph) were analysed by ELISA **(E)**.

As could be expected, we found concentrations of IL-12p40, IL-12p70, and IL-18 to significantly increase following stimulation of monocytes with R848 (Fig 4A). A role for these cytokines in monocyte-mediated stimulation of NK cell activity was furthermore confirmed in blocking experiments as we found anti-IL-12 as well as anti-IL-18 alone or in combination to significantly reduce monocyte-induced activation of NK cells (Fig 4B).

More importantly, we observed concentrations of IL-12p40 and IL-12p70, respectively, in supernatants of R848-stimulated monocytes to be associated with the *IL-28B* genotype. Monocytes from HCV(+) patients carrying a T/T genotype displayed the lowest IL-12 production (Fig 4C). When IL-18 production was analysed, we found that carriage of a C/T genotype was associated with highest concentrations whereas monocytes from T/T patients displayed the lowest IL-18 production. However, these differences were significant only when the T/T genotype was compared to carriage of the C-allele (Fig 4C).

To further confirm differential cytokine secretion to be involved in *IL-28B* genotype-dependent monocyte/NK cell interactions we next compared monocyte-induced NK cell IFN-γ production between carriers of different *IL-28B* genotypes following blockade of IL-12 and/or IL-18. As is depicted in Fig 4D, we found anti-IL-12-treatment to abrogate the association between *IL-28B* genotype and monocyte-induced NK cell production of IFN-γ. In contrast, monocytes of non-T/T patients remained more effective in triggering NK cell functions than monocytes of T/T patients even after blocking IL-18.

When healthy monocytes were studied, we found secretion of IL-12 and IL-18, respectively, to be independent of the *IL-28B* genotype (Fig 4E).

## Discussion

Epidemiologic studies suggested variants in close proximity to λ-IFN encoding genes to modulate NK cell functions [12,13]. However, the mechanisms underlying this association have remained unclear.

Here, we show that the extent of monocyte-induced and IL-12-mediated stimulation of NK cell IFN-γ production is associated with the *IL-28B* polymorphism in chronic hepatitis C, thereby providing a functional link between λ-IFNs and NK cell activity, which was independent on potentially confounding factors such as viral load or transaminase levels

IFN-λR1, the natural receptor of λ-IFNs, has been shown to be expressed primarily on epithelium-like tissues and, thus, leukocytes such as NK cells are considered not to respond to IFN-λ like IL-28A, IL-28B and IL-29 [15,23,24]. Accordingly, we and others demonstrated NK cells to be insensitive to stimulation with λ-IFNs [15,16], which could be confirmed in the present study.

Expression of IFN-α and IFN-γ receptors, respectively, has been shown to be upregulated following stimulation [25,26]. Therefore, we tested whether a similar mechanism may be relevant with respect to IFN-λR1. However, pre-stimulation of purified NK cell or total PBMC did not render NK cells susceptible towards λ-IFN stimulation, indicating that neither resting nor pre-activated NK cells respond to stimulation with IFN- λ.

Therefore, we hypothesized whether a rather indirect link might exist between *IL-28B* genotype and NK cell functions. To test this hypothesis in an experimental setting that mimics natural HCV infection, we stimulated cells with the TLR-7 agonist R848, as HCV RNA motifs have been shown to be recognized by TLR-7 [27]. Indeed, we observed a significant association between IFN-γ production of NK cells and the *IL-28B* polymorphism with carriers of a T/T genotype displaying the lowest frequency of IFN-γ (+) NK cells. Similar observations were made following stimulation with the TLR3 ligand Poly I/C. Of note, this association was only observed when total PBMC were stimulated with R848, whereas purified NK cells did not respond to R848, confirming earlier findings by Hart et al. [28]. Thus, we speculated that accessory cells might play an essential role in the activation of R848-mediated NK cell IFN-γ production. In line with this hypothesis, we found monocytes of *IL-28B* T/T patients to be significantly less effective in triggering IFN-γ production of NK cells than monocytes from carriers of a C/T and C/C genotype, respectively. Further experiments indicated that differential secretion of IL-12 and IL-18, which are known as potent stimulators of NK cells [28,29] importantly contributes to *IL-28B*-dependent stimulation of NK cell function by monocytes. First, we found R848-induced monocyte IL-12 secretion to be significantly lower in carriers of an *IL-28B* T/T genotype than in patients carrying a non-T/T variant. Second, we observed that blocking IL-12 but not IL-18 abrogates the *IL-28B* genotype dependent differences in monocyte-induced NK cell activation. Moreover, an association between the *IL-28B* polymorphism and cytokine levels has been reported before [30–32]. However, blocking of either IL-12 or IL-18 did not completely abrogate differential IFN-g production of NK cells from patients carrying different *IL28B* genotypes, suggesting that factors other than IL-12/IL-18 also might play a role in this context.

More recently, Mei and colleagues demonstrated an association between the *IFNL4* polymorphisms and IFN-γ response of PBMCs following TLR4 (LPS) and TLR7/8(R848) stimulation [33]. Of note, this TT/-G genetic variant has been shown to influences IL28B mRNA expression. Moreover, the ss469415590 (TT or ΔG) is in high linkage disequilibrium with rs12979860. Accordingly, in our cohort the ss469415590 [ΔG] allele was perfectly correlated with the unfavourable *IL28B* rs12979860[T] allele. Thus, we cannot exclude a functional role of the *IFNL4* polymorphisms in our setting.

In contrast to our study, Rogalska-Taranta et al. found that in patients with chronic hepatitis C carriage of an *IL-28B* T/T genotype was associated with high frequency of IFN-γ+ NK cells compared to carriers of an *IL-28B* C/C genotype, whereas the opposite was found in healthy controls [34]. These discrepant findings might be explained by a different study design as these authors directly stimulated PBMC-derived NK cells with recombinant IFN-α, whereas we used a TLR-7 agonist, which stimulates NK cells indirectly via activated monocytes. In line with our study, Jouvin-Marche et al. demonstrated a higher degranulation activity in liver NK cells/NKT cells of hepatitis C virus infected patients to be associated with *IL-28B* CC genotype in comparison to the unfavorable genotype [35]. Furthermore, Par et al. were able to show that chronic HCV patients achieving rapid virological response on PEG-IFN/ribavirin therapy showed an enhanced baseline proinflammatory cytokine production by TLR-4 activated monocytes compared to non-responder patients [36].

An important question relates to the mechanism underlying *IL-28B*-dependent IL-12 secretion by monocytes.

The *IL-28B* gene is located on chromosome 19 whereas the genes encoding IL-12A and IL-12B map to chromosomes 3 and 5, respectively. Thus, a strong genetic linkage seems rather unlikely. Moreover, expression of TLR-7 and TLR-8, the receptors for R848, did not differ between *IL-28B* genotypes.

Increased expression of interferon stimulated genes (ISGs) is a typical feature of chronic hepatitis C, most likely reflecting permanent stimulation by type I and/or type III IFNs [37]. Of note, the *IL-28B* genetic variant has repeatedly been shown to be associated with differential expression of ISGs [38–40]. In these studies, liver tissue from patients with chronic hepatitis C carrying the detrimental T/T genotype have been found to display significantly higher expression of ISGs compared to patients with a non-T/T genotype. This was an unexpected finding as the T/T genotype has been shown to be associated with low rates of spontaneous clearance of acute hepatitis C and poor response to IFN-α based therapy [5].

A possible explanation might be a setting in which *IL-28B* T/T genotype-associated up-regulation of ISGs in chronic hepatitis C results in exhaustion of the common pathway subsequently leading to ineffective response to type-I IFN [38]. In line with this hypothesis, Sung et al. recently demonstrated that expression of unphosphorylated ISG-F3 is increased by endogenous IFN in HCV-infected livers, leading to persistent activation of a set of ISGs and a lack of response to IFN-α in chronic HCV infection [41].

Since stimulation of TLR-7 has also been shown to induce expression of ISGs [42] a similar scenario of exhaustion might be envisioned for TLR-7-mediated signals, thereby explaining our finding that monocyte IL-12 production is impaired following stimulation with R848 in patients chronically infected with hepatits C virus who carry the *IL-28B* T/T genotype. Of note, an association between monocyte IL-12 production and *IL-28B* genotype was observed only in patients with chronic hepatitis C but not in healthy individuals. Again this would be compatible with the model of *IL-28B* genotype-dependent induction of ISGs because chronically upregulated IFN expression is considered to be essential for the observed ISG up-regulation in carriers of a T/T genotype in hepatitis C infection [43].

On the other hand, ISG expression in macrophages might be different from that observed in hepatocytes. Indeed, in Kupffer cells, the liver resident macrophages, the pre-therapy ISG levels have been shown to be opposite of those seen in hepatocytes, with patients not responding to IFN-α based treatment lacking induction of ISGs, whereas responders displayed strongly induced ISG [40]. Of note, high expression of the macrophage activation marker CD163 and the ISG MxA have been found to be associated with carriage of a C/C genotype [43,44], suggesting an *IL-28B* genotype-dependent activation of macrophages/Kupffer cells to modulate anti-HCV immune-responses. Thus, it is tempting to speculate that a similar mechanism might play a role in monocytes. In line with this hypothesis, cross-culture experiments demonstrated that *IL-28B* genotype dependent IFN-γ production of NK cells was mainly attributable to increased IL-12 production of HCV(+) monocytes. Moreover, we observed higher expression of pSTAT1 and pSTAT5 in monocytes from patients with a non-T/T genotype than in carriers of a T/T genotype. However, these differences did not reach statistical significance.

Taken together, we show that in chronic hepatitis C the extent of monocyte-induced and IL-12-mediated stimulation of NK cell IFN-γ production is associated with the *IL-28B* rs12979860 polymorphism, thereby providing a mechanistic link between NK cell function and the *IL-28B* genotype.

## Statement

In a poster abstract presented on the “Liver meeting 2015” of AASLD with the title “*IL-28B* genetic variants determine the extent of monocyte-induced activation of NK cells in hepatitis C”we reported that a total of 74 HCV(+) patients and 80 healthy controls were enrolled into the study. However, numbers of participants in the present publication are different. The poster abstract had been erroneously included participant samples from preliminary experiments with different workflow.

## Supporting Information

S1 Fig Exemplary gating strategy for PBMC-derived NK cells analyzed by flow cytometry. CD56+CD3- NK cells were sub-divided in CD56Bright und CD56 Dim NK cells by CD56/CD16 gating (A). PBMC from HCV patients were pre-stimulated with R848 then co-cultured with HUH7HCVreplicon cells (B) or hepatic stellate cells (C), respectively. After 5h of co-incubation IFN-γ production (B) or degranulation (C) of NK cells was studied by FACS analysis, respectively. The exemplary histograms show IFN-γ production (B) or degranulation (C) of CD56Bright (left side) and CD56Dim NK cells (right side) from HCV patients with different *IL28-B* genotypes (Non-TT vs TT), respectively.
(PDF)

S2 Fig Monocyte-induced NK cell activation by TLR3 ligand Poly I/C is associated with the *IL-28B* genotype. Monocytes from HCV patients were pre-stimulated with Poly-I/C and then co-cultured with autologous NK cells in the HUH7HCVreplicon cells. After 5h of co-incubation IFN-γ production of NK cells was studied by FACS analysis. This figure shows IFN-γ production of NK cells from HCV patients with different *IL-28B* genotypes (CC vs. TC vs. TT; * P<0.05).
(PDF)

S3 Fig Cross-coculture experiments with monocyte/NK cells from healthy and HCV infected subjects. Monocytes from HCV patients (A) were pre-stimulated with R848 then co-cultured with healthy NK cells in the HUH7HCVreplicon cells and vice versa (B). After 5h of co-incubation IFN-γ production of NK cells was studied by FACS analysis. This figure shows IFN-γ production of NK cells from healthy donors (A) or HCV patients (B) with different *IL-28B* genotypes (CC vs. TC vs. TT; * P<0.05; n.s. not significant).
(PDF)

S4 Fig Serum alanine aminotransferase levels and HCV viral load have no impact on NK cell IFN-γ production in HCV infected persons. Total PBMCs from HCV patients with different *IL-28B* genotypes (Non-TT, n = 20; T/T, n = 7) were pre-stimulated with R848 then co-cultured with HUH7HCVreplicon cells. After 5h of co-incubation IFN-γ production of CD56Bright NK cells was studied by FACS analysis. The figure shows the IFN-γ production of CD56Bright NK cells depending on serum alanine aminotransferase (A: ALT <40 vs. <40 and >120 vs. >120 U/l) and HCV viral load(B: HCV viral load <8x105 vs. >8x105 IU/ml; n.s. not significant).
(PDF)

S1 Table Raw data of Figs 1–4 and clinical data. This table includes all raw data of Figs 1–4 and the patients’ characteristics (clinical data).
(PDF)

## Abbreviations

E:T ratio: effector:target ratio
FACS: fluorescence-activated cell sorting
HCV: hepatitis C virus
HSC: hepatic stellate cells
IFN: interferon
IFN-λR1: interferon lambda receptor 1
IL: interleukin
KIR: killer cell immunoglobulin-like receptors
NK cell: natural killer cell
PBMC: peripheral blood mononuclear cells
rtPCR: real-time polymerase chain reaction
SNP: single nucleotide polymorphism
TLR: toll-like receptors

## References

[1] Rehermann B. Pathogenesis of chronic viral hepatitis: differential roles of T cells and NK cells. Nat Med. 2013;19: 859–868. 10.1038/nm.3251 23836236 PMC4482132

[2] Matsuura K, Tanaka Y. Host genetic variants influencing the clinical course of hepatitis C virus infection. J Med Virol. 2016;88: 185–195. 10.1002/jmv.24334 26211651

[3] Suppiah V, Moldovan M, Ahlenstiel G, Berg T, Weltman M, Abate ML, et al IL28B is associated with response to chronic hepatitis C interferon-alpha and ribavirin therapy. Nat Genet. 2009;41: 1100–1104. 10.1038/ng.447 19749758

[4] Rauch A, Kutalik Z, Descombes P, Cai T, Di Iulio J, Mueller T, et al Genetic variation in IL28B is associated with chronic hepatitis C and treatment failure: a genome-wide association study. Gastroenterology. 2010;138: 1338–1345, 1345–7. 10.1053/j.gastro.2009.12.056 20060832

[5] Tanaka Y, Nishida N, Sugiyama M, Kurosaki M, Matsuura K, Sakamoto N, et al Genome-wide association of IL28B with response to pegylated interferon-alpha and ribavirin therapy for chronic hepatitis C. Nat Genet. 2009;41: 1105–1109. 10.1038/ng.449 19749757

[6] Prokunina-Olsson L, Muchmore B, Tang W, Pfeiffer RM, Park H, Dickensheets H, et al A variant upstream of IFNL3 (IL28B) creating a new interferon gene IFNL4 is associated with impaired clearance of hepatitis C virus. Nat Genet. 2013; 10.1038/ng.2521 PMC3793390 23291588

[7] Bibert S, Roger T, Calandra T, Bochud M, Cerny A, Semmo N, et al IL28B expression depends on a novel TT/-G polymorphism which improves HCV clearance prediction. J Exp Med. 2013;210: 1109–1116. 10.1084/jem.20130012 23712427 PMC3674704

[8] Khakoo SI, Thio CL, Martin MP, Brooks CR, Gao X, Astemborski J, et al HLA and NK cell inhibitory receptor genes in resolving hepatitis C virus infection. Science. 2004;305: 872–874. 10.1126/science.1097670 15297676

[9] Knapp S, Warshow U, Hegazy D, Brackenbury L, Guha IN, Fowell A, et al Consistent beneficial effects of killer cell immunoglobulin-like receptor 2DL3 and group 1 human leukocyte antigen-C following exposure to hepatitis C virus. Hepatol Baltim Md. 2010;51: 1168–1175. 10.1002/hep.23477 PMC4202114 20077564

[10] Vidal-Castiñeira JR, López-Vázquez A, Díaz-Peña R, Alonso-Arias R, Martínez-Borra J, Pérez R, et al Effect Of Killer Immunoglobulin-Like Receptors (Kir) in the Response to Combined Treatment In Patients With Chronic Hepatitis C. J Virol. 2009; 10.1128/JVI.01285-09 PMC2798445 19846535

[11] Ahlenstiel G, Martin MP, Gao X, Carrington M, Rehermann B. Distinct KIR/HLA compound genotypes affect the kinetics of human antiviral natural killer cell responses. J Clin Invest. 2008; 10.1172/JCI32400 PMC2214845 18246204

[12] Dring MM, Morrison MH, McSharry BP, Guinan KJ, Hagan R, O’Farrelly C, et al Innate immune genes synergize to predict increased risk of chronic disease in hepatitis C virus infection. Proc Natl Acad Sci. 2011;108: 5736–5741. 10.1073/pnas.1016358108 21402922 PMC3078345

[13] Suppiah V, Gaudieri S, Armstrong NJ, O’Connor KS, Berg T, Weltman M, et al IL28B, HLA-C, and KIR Variants Additively Predict Response to Therapy in Chronic Hepatitis C Virus Infection in a European Cohort: A Cross-Sectional Study. PLoS Med. 2011;8: e1001092 10.1371/journal.pmed.1001092 21931540 PMC3172251

[14] Souza-Fonseca-Guimaraes F, Young A, Mittal D, Martinet L, Bruedigam C, Takeda K, et al NK cells require IL-28R for optimal in vivo activity. Proc Natl Acad Sci. 2015;112: E2376–E2384. 10.1073/pnas.1424241112 25901316 PMC4426428

[15] Krämer B, Eisenhardt M, Glässner A, Körner C, Sauerbruch T, Spengler U, et al Do λ-IFNs IL28A and IL28B act on human natural killer cells? Proc Natl Acad Sci. 2011;108: E519–E520. 10.1073/pnas.1108850108 21825163 PMC3161529

[16] Morrison MH, Keane C, Quinn LM, Kelly A, O’Farrelly C, Bergin C, et al IFNL cytokines do not modulate human or murine NK cell functions. Hum Immunol. 2014;75: 996–1000. 10.1016/j.humimm.2014.06.016 24994459

[17] Binder M, Quinkert D, Bochkarova O, Klein R, Kezmic N, Bartenschlager R, et al Identification of determinants involved in initiation of hepatitis C virus RNA synthesis by using intergenotypic replicase chimeras. J Virol. 2007;81: 5270–5283. 10.1128/JVI.00032-07 17344294 PMC1900214

[18] Krämer B, Körner C, Kebschull M, Glässner A, Eisenhardt M, Nischalke H-D, et al Natural killer p46High expression defines a natural killer cell subset that is potentially involved in control of hepatitis C virus replication and modulation of liver fibrosis. Hepatol Baltim Md. 2012;56: 1201–1213. 10.1002/hep.25804 22532190

[19] Wang N, Liang Y, Devaraj S, Wang J, Lemon SM, Li K. Toll-Like Receptor 3 Mediates Establishment of an Antiviral State against Hepatitis C Virus in Hepatoma Cells. J Virol. 2009;83: 9824–9834. 10.1128/JVI.01125-09 19625408 PMC2747996

[20] Larangé A, Antonios D, Pallardy M, Kerdine-Römer S. TLR7 and TLR8 agonists trigger different signaling pathways for human dendritic cell maturation. J Leukoc Biol. 2009;85: 673–683. 10.1189/jlb.0808504 19164127

[21] Arima K, Watanabe N, Hanabuchi S, Chang M, Sun S-C, Liu Y-J. Distinct Signal Codes Generate Dendritic Cell Functional Plasticity. Sci Signal. 2010;3: ra4 10.1126/scisignal.2000567 20086239 PMC3325779

[22] Fehniger TA, Shah MH, Turner MJ, VanDeusen JB, Whitman SP, Cooper MA, et al Differential Cytokine and Chemokine Gene Expression by Human NK Cells Following Activation with IL-18 or IL-15 in Combination with IL-12: Implications for the Innate Immune Response. J Immunol. 1999;162: 4511–4520. 10201989

[23] Sommereyns C, Paul S, Staeheli P, Michiels T. IFN-Lambda (IFN-λ) Is Expressed in a Tissue-Dependent Fashion and Primarily Acts on Epithelial Cells In Vivo. PLoS Pathog. 2008;4: e1000017 10.1371/journal.ppat.1000017 18369468 PMC2265414

[24] Dickensheets H, Sheikh F, Park O, Gao B, Donnelly RP. Interferon-lambda (IFN-λ) induces signal transduction and gene expression in human hepatocytes, but not in lymphocytes or monocytes. J Leukoc Biol. 2013;93: 377–385. 10.1189/jlb.0812395 23258595 PMC3579021

[25] Shirey KA, Jung J-Y, Maeder GS, Carlin JM. Upregulation of IFN-gamma receptor expression by proinflammatory cytokines influences IDO activation in epithelial cells. J Interferon Cytokine Res Off J Int Soc Interferon Cytokine Res. 2006;26: 53–62. 10.1089/jir.2006.26.53 PMC1550344 16426148

[26] Mizukoshi E, Kaneko S, Yanagi M, Ohno H, Matsushita E, Kobayashi K. Upregulation of type I interferon receptor by IFN-gamma. J Interferon Cytokine Res Off J Int Soc Interferon Cytokine Res. 1999;19: 1019–1023. 10.1089/107999099313235 10505744

[27] Zhang Y-L, Guo Y-J, Bin Li null, Sun S-H. Hepatitis C virus single-stranded RNA induces innate immunity via Toll-like receptor 7. J Hepatol. 2009;51: 29–38. 10.1016/j.jhep.2009.03.012 19443072

[28] Hart OM, Athie-Morales V, O’Connor GM, Gardiner CM. TLR7/8-mediated activation of human NK cells results in accessory cell-dependent IFN-gamma production. J Immunol Baltim Md 1950. 2005;175: 1636–1642. 10.4049/jimmunol.175.3.1636 16034103

[29] Serti E, Werner JM, Chattergoon M, Cox AL, Lohmann V, Rehermann B. Monocytes activate natural killer cells via inflammasome-induced interleukin 18 in response to hepatitis C virus replication. Gastroenterology. 2014;147: 209–220.e3. 10.1053/j.gastro.2014.03.046 24685721 PMC4469643

[30] de Sá KSG, Santana BB, de Souza Ferreira TC, Sousa RCM, Caldas CAM, Azevedo VN, et al IL28B gene polymorphisms and Th1/Th2 cytokine levels might be associated with HTLV-associated arthropathy. Cytokine. 2016;77: 79–87. 10.1016/j.cyto.2015.11.004 26546777

[31] Naggie S, Osinusi A, Katsounas A, Lempicki R, Herrmann E, Thompson AJ, et al Dysregulation of innate immunity in hepatitis C virus genotype 1 IL28B-unfavorable genotype patients: Impaired viral kinetics and therapeutic response. Hepatology. 2012;56: 444–454. 10.1002/hep.25647 22331604 PMC3361636

[32] Umemura T, Joshita S, Yoneda S, Katsuyama Y, Ichijo T, Matsumoto A, et al Serum interleukin (IL)-10 and IL-12 levels and IL28B gene polymorphisms: pretreatment prediction of treatment failure in chronic hepatitis C. Antivir Ther. 2011;16: 1073–1080. 10.3851/IMP1869 22024523

[33] Chen Yi Mei SLG, Burchell J, Skinner N, Millen R, Matthews G, Hellard M, et al Toll-like Receptor Expression and Signaling in Peripheral Blood Mononuclear Cells Correlate With Clinical Outcomes in Acute Hepatitis C Virus Infection. J Infect Dis. 2016; 10.1093/infdis/jiw235 PMC4978374 27284092

[34] Rogalska-Taranta M, Markova AA, Taranta A, Lunemann S, Schlaphoff V, Flisiak R, et al Altered effector functions of NK cells in chronic hepatitis C are associated with IFNL3 polymorphism. J Leukoc Biol. 2015;98: 283–294. 10.1189/jlb.4A1014-520R 26034208

[35] Jouvin-Marche E, Jílková ZM, Thelu M-A, Marche H, Fugier E, Campenhout NV, et al Lymphocytes Degranulation in Liver in Hepatitis C Virus Carriers Is Associated With IFNL4 Polymorphisms and ALT Levels. J Infect Dis. 2014;209: 1907–1915. 10.1093/infdis/jiu016 24415789

[36] Par G, Szereday L, Berki T, Palinkas L, Halasz M, Miseta A, et al Increased Baseline Proinflammatory Cytokine Production in Chronic Hepatitis C Patients with Rapid Virological Response to Peginterferon Plus Ribavirin. PLOS ONE. 2013;8: e67770 10.1371/journal.pone.0067770 23874444 PMC3706447

[37] Metz P, Reuter A, Bender S, Bartenschlager R. Interferon-stimulated genes and their role in controlling hepatitis C virus. J Hepatol. 2013;59: 1331–1341. 10.1016/j.jhep.2013.07.033 23933585

[38] Urban TJ, Thompson AJ, Bradrick SS, Fellay J, Schuppan D, Cronin KD, et al IL28B genotype is associated with differential expression of intrahepatic interferon-stimulated genes in patients with chronic hepatitis C. Hepatology. 2010;52: 1888–1896. 10.1002/hep.23912 20931559 PMC3653303

[39] Honda M, Shirasaki T, Shimakami T, Sakai A, Horii R, Arai K, et al Hepatic interferon-stimulated genes are differentially regulated in the liver of chronic hepatitis C patients with different interleukin-28B genotypes. Hepatol Baltim Md. 2014;59: 828–838. 10.1002/hep.26788 24311440

[40] Chen L, Borozan I, Sun J, Guindi M, Fischer S, Feld J, et al Cell-Type Specific Gene Expression Signature in Liver Underlies Response to Interferon Therapy in Chronic Hepatitis C Infection. Gastroenterology. 2010;138: 1123–1133.e3. 10.1053/j.gastro.2009.10.046 19900446

[41] Sung PS, Cheon H, Cho CH, Hong S-H, Park DY, Seo H-I, et al Roles of unphosphorylated ISGF3 in HCV infection and interferon responsiveness. Proc Natl Acad Sci. 2015;112: 10443–10448. 10.1073/pnas.1513341112 26216956 PMC4547285

[42] Puig M, Tosh KW, Schramm LM, Grajkowska LT, Kirschman KD, Tami C, et al TLR9 and TLR7 agonists mediate distinct type I IFN responses in humans and nonhuman primates in vitro and in vivo. J Leukoc Biol. 2012;91: 147–158. 10.1189/jlb.0711371 22058422

[43] McGilvray I, Feld JJ, Chen L, Pattullo V, Guindi M, Fischer S, et al Hepatic Cell–Type Specific Gene Expression Better Predicts HCV Treatment Outcome Than IL28B Genotype. Gastroenterology. 2012;142: 1122–1131.e1. 10.1053/j.gastro.2012.01.028 22285807

[44] Dultz G, Gerber L, Zeuzem S, Sarrazin C, Waidmann O. The macrophage activation marker CD163 is associated with IL28B genotype and hepatic inflammation in chronic hepatitis C virus infected patients. J Viral Hepat. 2015; n/a-n/a. 10.1111/jvh.12488 26554542

