# Supplementary material for: IL-28B Genetic Variants Determine the Extent of Monocyte-Induced Activation of NK Cells in Hepatitis C
Source: PLoS One. 2016 Sep 1;11(9):e0162068. doi: 10.1371/journal.pone.0162068 (PMC5008784; doi:10.1371/journal.pone.0162068)
Supplement: S1 Fig — CD56+CD3- NK cells were sub-divided in CD56Bright und CD56 Dim NK cells by CD56/CD16 gating (A). PBMC from HCV patients were pre-stimulated with R848 then co-cultured with HUH7HCVreplicon cells (B) or hepatic stellate cells (C), respectively. After 5h of co-incubation IFN-γ production (B) or degranulation (C) of NK cells was studied by FACS analysis, respectively. The exemplary histograms show IFN-γ production (B) or degranulation (C) of CD56Bright (left side) and CD56Dim NK cells (right side) from HCV patients with different IL28-B genotypes (Non-TT vs TT), respectively. (PDF) [file pone.0162068.s001.pdf]

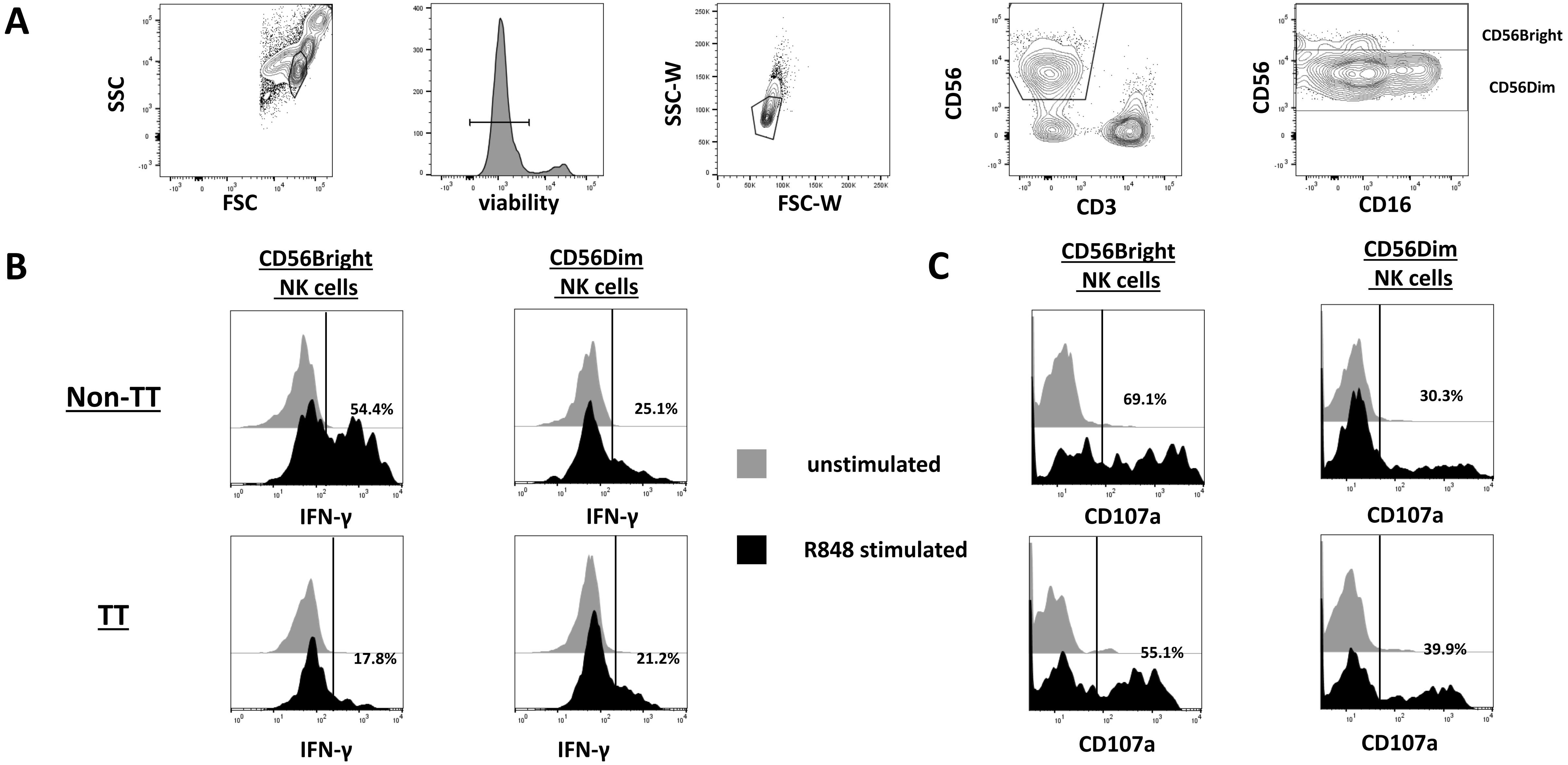

Supplemental Figure 1: **Exemplary gating strategy for PBMC-derived NK cells analyzed by flow cytometry.** CD56+CD3- NK cells were sub-divided in CD56Bright und CD56 Dim NK cells by CD56/CD16 gating (A). PBMC from HCV patients were pre-stimulated with R848 then co-cultured with HUH7HCVreplicon cells(B) or Hepatic stellate cells (C), respectively. After 5h of co-incubation IFN-γ production (B) or degranulation (C) of NK cells was studied by FACS analysis, respectively. The exemplary histograms show IFN-γ production (B) or degranulation (C) of CD56Bright (left side) and CD56Dim NK cells (right side) from HCV patients with different *IL-28B* genotypes (Non-TT vs TT), respectively.
