# Supplementary material for: IL-28B Genetic Variants Determine the Extent of Monocyte-Induced Activation of NK Cells in Hepatitis C
Source: PLoS One. 2016 Sep 1;11(9):e0162068. doi: 10.1371/journal.pone.0162068 (PMC5008784; doi:10.1371/journal.pone.0162068)
Supplement: S2 Fig — Monocytes from HCV patients were pre-stimulated with Poly-I/C and then co-cultured with autologous NK cells in the HUH7HCVreplicon cells. After 5h of co-incubation IFN-γ production of NK cells was studied by FACS analysis. This figure shows IFN-γ production of NK cells from HCV patients with different IL-28B genotypes (CC vs. TC vs. TT; * P<0.05). (PDF) [file pone.0162068.s002.pdf]

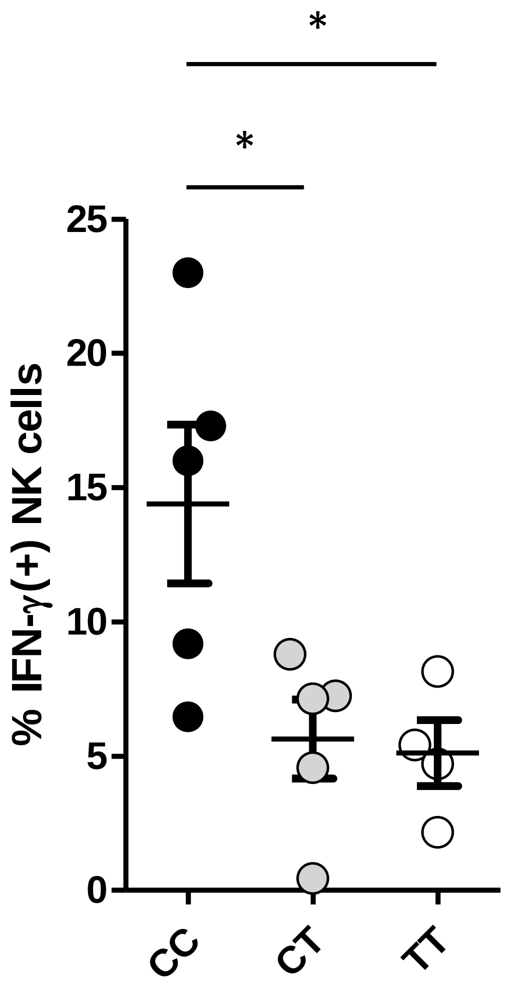

Supplemental Figure 2: **Monocyte-induced NK cell activation by TLR3 ligand Poly I/C is also associated with the IL-28B genotype.** Monocytes from HCV patients were pre-stimulated with Poly-I/C then co-cultured with autolog NK cells in the HUH7HCVreplicon cells. After 5h of co-incubation IFN-γ production of NK cells was studied by FACS analysis. This Figure shows IFN-γ production of NK cells from HCV patients with different *IL-28B* genotypes (CC vs. TC vs. TT; * $P<0.05$).
