# Supplementary material for: IL-28B Genetic Variants Determine the Extent of Monocyte-Induced Activation of NK Cells in Hepatitis C
Source: PLoS One. 2016 Sep 1;11(9):e0162068. doi: 10.1371/journal.pone.0162068 (PMC5008784; doi:10.1371/journal.pone.0162068)
Supplement: S3 Fig — Monocytes from HCV patients (A) were pre-stimulated with R848 then co-cultured with healthy NK cells in the HUH7HCVreplicon cells and vice versa (B). After 5h of co-incubation IFN-γ production of NK cells was studied by FACS analysis. This figure shows IFN-γ production of NK cells from healthy donors (A) or HCV patients (B) with different IL-28B genotypes (CC vs. TC vs. TT; * P<0.05; n.s. not significant). (PDF) [file pone.0162068.s003.pdf]

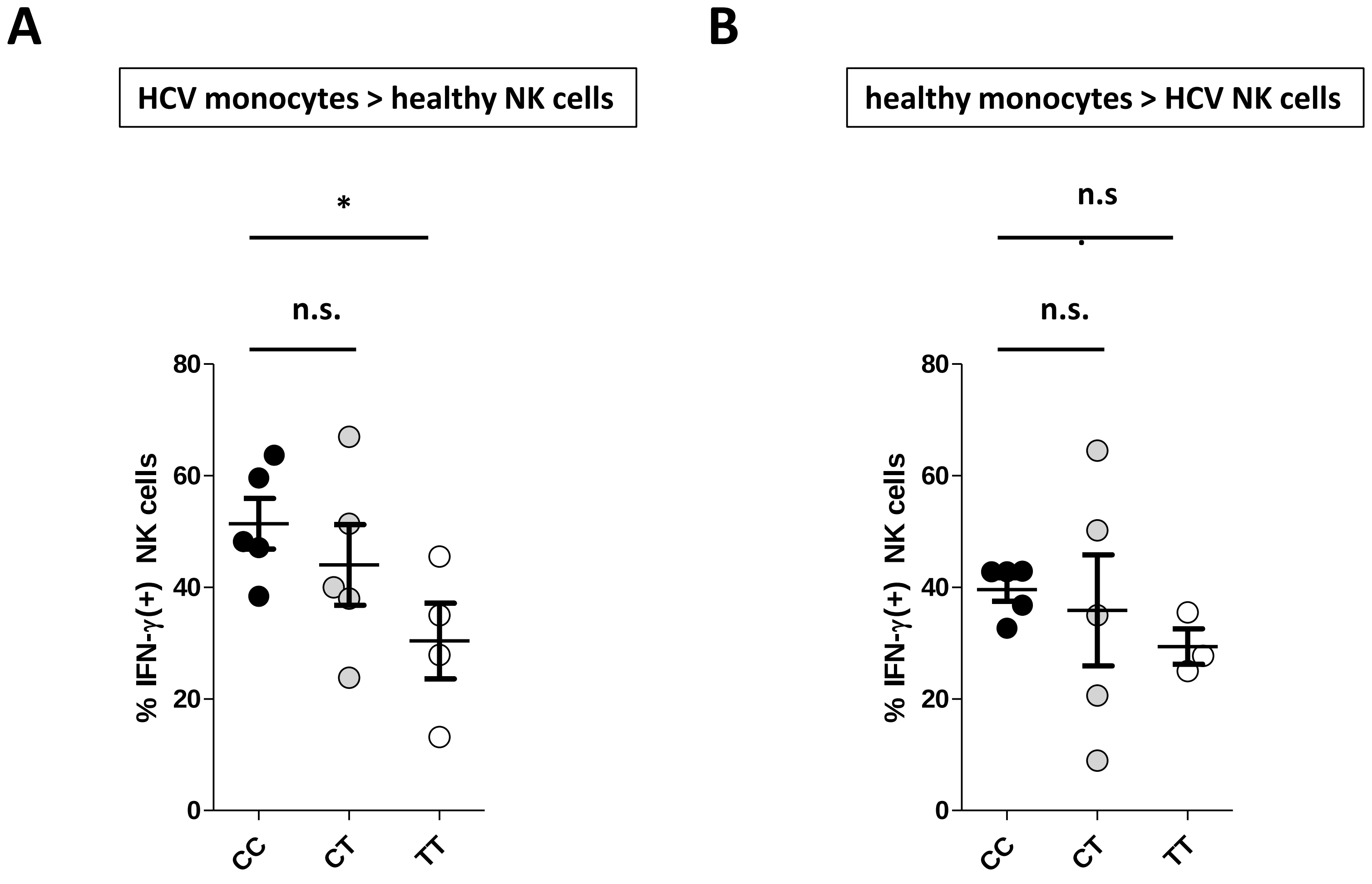

Supplemental Figure 3: **Cross-coculture experiments with monocyte/NK cells from healthy and HCV infected subjects.** Monocytes from HCV patients (A) were pre-stimulated with R848 then co-cultured with healthy NK cells in the HUH7HCVreplicon cells and vice versa (B). After 5h of co-incubation IFN-γ production of NK cells was studied by FACS analysis. This Figure shows IFN-γ production of NK cells from healthy donors (A) or HCV patients (B) with different *IL-28B* genotypes (CC vs. TC vs. TT ; * P<0.05; n.s. not significant).
