# Supplementary material for: IL-28B Genetic Variants Determine the Extent of Monocyte-Induced Activation of NK Cells in Hepatitis C
Source: PLoS One. 2016 Sep 1;11(9):e0162068. doi: 10.1371/journal.pone.0162068 (PMC5008784; doi:10.1371/journal.pone.0162068)
Supplement: S4 Fig — Total PBMCs from HCV patients with different IL-28B genotypes (Non-TT, n = 20; T/T, n = 7) were pre-stimulated with R848 then co-cultured with HUH7HCVreplicon cells. After 5h of co-incubation IFN-γ production of CD56Bright NK cells was studied by FACS analysis. The figure shows the IFN-γ production of CD56Bright NK cells depending on serum alanine aminotransferase (A: ALT <40 vs. <40 and >120 vs. >120 U/l) and HCV viral load(B: HCV viral load <8x10^5^ vs. >8x10^5^ IU/ml; n.s. not significant). (PDF) [file pone.0162068.s004.pdf]

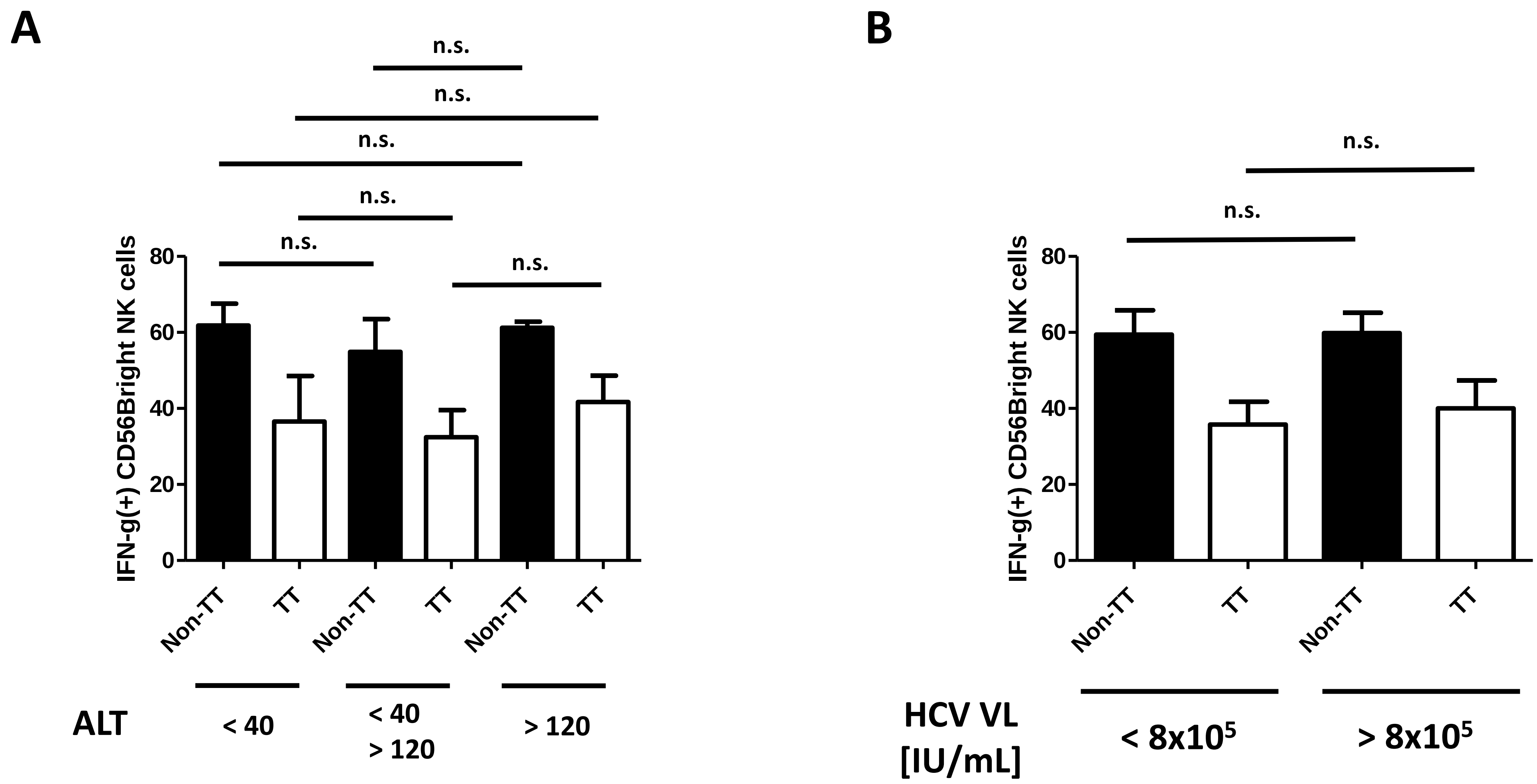

Supplemental Figure 4: **Serum alanine aminotransferase levels and HCV viral load have no impact on NK cell IFN-γ production in HCV infected persons.** Total PBMCs from HCV patients with different *IL-28B* genotypes (Non-TT, n=20; T/T, n=7) were pre-stimulated with R848 then co-cultured with HUH7HCVreplicon cells. After 5h of co-incubation IFN-γ production of CD56Bright NK cells was studied by FACS analysis. The figure shows the IFN-γ production of CD56Bright NK cells depending on serum alanine aminotransferase (**A**: ALT <40 **vs.** <40 and >120 **vs.** >120 U/l) and HCV viral load(**B**: HCV viral load <8x10^5 **vs**. >8x10^5 IU/ml; n.s. not significant).
