# Supplementary material for: IL-28B Genetic Variants Determine the Extent of Monocyte-Induced Activation of NK Cells in Hepatitis C
Source: PLoS One. 2016 Sep 1;11(9):e0162068. doi: 10.1371/journal.pone.0162068 (PMC5008784; doi:10.1371/journal.pone.0162068)
Supplement: S1 Table — This table includes all raw data of Figs 1–4 and the patients’ characteristics (clinical data). (PDF) [file pone.0162068.s005.pdf]

S1 Table: Raw data of Fig 1-4 and clinical data. This table includes all raw data of Fig 1-4 and the patients' characteristics (clinical data).

Fig. 1

Fig. 1A

IL28B CC unstim	IL28B CC IL29	IL28B CT unstim	IL28B CT IL29	IL28B TT unstim	IL28B TT IL29
0,956	0,82	0,704	0,713	2,56	1,21
0,858	0,181	1,27	0,828	0,674	1,27
0,8	1,4	0,847	2,28	2,01	2,63
0,545	1,26	2,6	2,02	1,45	2,59
0,227	6,6	0	0,197	4,32	3,52
0,412	0,887	0,683	0,543	1,67	2,4
2,8	1,77	4,44	2,4	0,405	0,626
0,266	0,552	2,36	0,804		
		1,13	0,888		
		3,06	2,27		
		1,13	1,79		
		0,175	0,12		

Fig. 1B

unstim	IL29	R848	R848+IL29
0	0,197	33,2	33,3
0,175	0,12	45,4	46,4
0,227	6,6	28,2	29
0,266	0,552	23,7	37,9
0,405	0,626	26	30,5
0,412	0,887	22,9	18,6
0,545	1,26	30,9	42,2
0,674	1,27	19,7	23,4
0,683	0,543	48,5	41
0,704	0,713	51,8	63,6
0,8	1,4	14,8	20,3
0,847	2,28	13,7	18,4
0,858	0,181	19,5	26,4
0,956	0,82	26,2	21,3
1,13	0,888	27,2	36,3
1,13	1,79	52,8	46,9
1,27	0,828	23,5	24,2
1,45	2,59	24,2	25,4
1,67	2,4	14,4	8,19
2,01	2,63	42	47
2,36	0,804	33,6	45,8
2,56	1,21	28,9	22,8
2,6	2,02	23,1	32,5
2,8	1,77	40,7	39
3,06	2,27	18	21,7
4,32	3,52	19,3	22,1
4,44	2,4	56,5	60,2

Fig. 2

Fig. 2A

HCV PBMC R848 stim

IL28B CC	IL28B CT	IL28B TT
64	73,6	25,3
56	54,7	24,6
62,5	23,2	46,1
68,2	23,6	28
76,7	62,6	48,6
60,7	64,8	39,6
62,4	90,2	50,9
59,3	58,2	
	41,5	
	37,1	
	63,2	
	90,9	

Fig. 2A

HCV PBMC R848 stim + IL29

IL28B CC	IL28B CT	IL28B TT
57,4	80,4	12,5
61,3	66,7	32,1
69,9	23,4	55,1
86,7	27,5	25
87,2	64,7	38,5
65,7	52,9	25
68,9	91,9	56,6
64,8	71,4	
	69	
	43,2	
	58,5	
	84,8	

Fig. 2B

HCV PBMC R848 stim

IL28B CC	IL28B CT	IL28B TT
64,3	46,8	5,36
71,2	54,3	55,8
69,5	63	3,83
42,9	33,2	56,4
50,8	47,5	12
37,2	50,8	43,2
69,1	68,2	44
	54,1	
	66	
	56,8	
	40,6	
	46,5	

Fig. 2B

HCV PBMC R848 stim + IL29

IL28B CC	IL28B CT	IL28B TT
44,7	43,6	5,05
76,2	56,9	57
66,4	63,2	4,58
55,5	36,8	60,3
52,2	43,5	16
48,7	55	44
75,3	66,1	40,7
	57,4	
	66,9	
	44,1	
	41,7	
	49,4	

Fig. 2C

HCV NK R848 stim

IL28B CC	IL28B CT	IL28B TT
1,94	0,26	0,17
2,34	0,27	0,49
2,8	0,56	1,42
5,6	1,23	2,43
7,4	2,56	8,01
9,2	3,63	14,4
9,99	7,56	
1,03	7,4	
4,05	8,5	
	12,4	

Fig. 2D

Healthy PBMC R848 stim

IL28B CC	IL28B CT	IL28B TT
47,1	33,3	56,8
39,6	31,1	63,6
50,6	34,3	54
38,9	34,8	50,8
80,2	36,4	33,3
49,3	61,1	
19,2	39,6	
55,6	61,2	
56,7	49,7	
64,5	46,8	
61,9	72,6	
68,1	26,7	
65,2	80,3	
73,3	38,8	
74,5	92,5	
60,7	96,1	
32,1	78,8	
81	52,8	
75,9	27,2	
26,7	22,4	
14		
76,3		
77,8		

Fig. 3

Fig. 3A

HCV Mo + NK R848 stim

IL28B CC	IL28B CT	IL28B TT
34,9	29,2	16,8
85,4	58,8	12,8
36,5	52,2	30,4
54,3	75,1	58,7
45,4	15,3	20
82,8	46,9	45,6
59,3	75,4	21,5
41,9	85,7	67
59,7	79,8	35,2
79,1		11,5
		49,7
		20,7

Fig. 3B

Healthy Mo + NK R848 stim

IL28B CC R848	IL28B CT R848	IL28B TT R848
59,1	57,9	75,1
60,7	28,1	40,8
59,7	57,9	76
42,6	39,4	25,8
	30	
	34,5	

Fig. 3C

HCV TLR7 Mono

unstim	R848
51,8	58,4
58,9	93,7
78,8	65,4
69,6	66,8
57,4	97,5
57,2	66
43,6	60,3
55,4	58,3
49,1	63,3

Fig. 3C

HCV TLR7 R848 Mono

Non-TT	TT
58,4	66
93,7	60,3
65,4	58,3
66,8	63,3
97,5	

Fig. 3D

HCV TLR8 Mono

unstim	R848
202	174
169	263
186	209
189	179
175	222
203	178
179	189
188	170
161	186

Fig. 3D

HCV TLR8 R848 Mono

Non-TT	TT
174	178
263	189
209	170
179	186
222	

Fig. 3E

HCV Stat1 R848 Mono

Non-TT	TT
125	67,8
76,1	81,4
120	88,8
60,1	25,8

Fig. 3E

HCV Stat5 R848 Mono

Non-TT	TT
12,5	10,1
9,87	11,7
16,2	10,5
9,28	5,85

Fig. 4

Fig. 4A

IL-12p70 unstim	IL-12p70 R848	IL-12p40 unstim	IL-12p40 R848	IL-18 unstim	IL-18 R848
0	36,3744	0,1596062	79,00001	5,128	144,0968
0	21,2184	2,7394	311,7518	6,41	158,1988
4,5468	43,9524	7,24964	9,15744	6,6664	135,3792
4,5468	16,6716	7,6312	119,8098	6,9228	119,7388
7,578	30,312	9,15744	289,604	7,1792	26,6656
9,0936	244,0116	9,539	136,5985	7,4356	157,4296
9,0936	1,5156	9,539	86,61412	7,4356	165,8908
9,0936	24,2496	9,92056	93,4822	7,4356	74,6124
10,6092	101,5452	10,68368	76,69356	7,9484	34,8704
12,1248	18,1872	11,4468	243,8168	8,2048	30,2552
16,6716	39,4056	11,82836	260,987	8,4612	32,05
19,7028	65,1708	13,73616	56,85244	9,7432	200,5048
		47,85918	232,9856	10,256	83,8428
		60,14596	334,5434	11,2816	13,5892
		61,1766	536,9946	12,3072	83,33
		72,5056	317,212		
		77,0372	212,9852		
		77,0372	305,883		
		83,54922	544,983		

Fig. 4B

unstim	R848 w/o Ab	R848+ Iso	R848+ anti-IL12	R848+ anti-IL12	R848+ anti-IL12+IL18
0	20	29,4	7,11	12,2	3,97
0	12,8	11,6	5,31	4,8	2,48
0	58,7	53	27,4	13,1	4,15
0	21,5	20,1	2,37	6,28	2,09
0,14	59,3	59,1	21,7	15,7	4,9
0,25	36,5	36,8	11,4	10,6	4,83
0,3	91,9	88,6	33	35,6	2,77
0,47	49,7	35,8	7,9	2,46	2,33
0,47	41,9	50,2	12,3	8,66	0,13
0,6	79,8	75,9	21,4	25,9	4,7
1,12	85,4	83,5	26	45,2	5,63
1,2	21,7	25,4	8,72	0,64	2,78
2,25	82,6	85,6	43,7	54,2	16,9
13	54,3	64,5	10,2	20	1,77
16,9	85,7	78,1	58,7	43,5	12,3
22,1	71,7	70,7	31,2	41,3	9,13

Fig. 4C

HCV IL-12p70 R848

IL28B CC	IL28B CT	IL28B TT
18,1872	1,5156	24,2496
36,3744	43,9524	30,312
39,4056	101,5452	21,2184
65,1708		16,6716
244,0116		

Fig. 4C

HCV IL-12p40 R848

IL28B CC	IL28B CT	IL28B TT
136,5985	76,69356	86,61412
212,9852	79,00001	93,4822
289,604	119,8098	9,15744
311,7518	232,9856	56,85244
334,5434	243,8168	
536,9946	260,987	
544,983	305,883	
	317,212	

Fig. 4C

HCV IL-18 R848

IL28B CC	IL28B CT	IL28B TT
30,2552	25,64	14,102
34,8704	157,4296	83,33
42,306	165,8908	74,6124
83,8428	200,5048	32,05
119,7388		
135,3792		
158,1988		

Fig. 4D

R848 IL28B CC	R848 IL28B CT	R848 IL28B TT	R848 Block IL12 IL28B CC	R848 Block IL12 IL28B CT	R848 Block IL12 IL28B TT	R848 Block IL18 IL28B CC	R848 Block IL18 IL28B CT	R848 Block IL18 IL28B TT	R848 Block IL12+IL18 IL28B CC	R848 Block IL12+IL18 IL28B CT	R848 Block IL12+IL18 IL28B TT
59,3	91,9	20	21,7	33	7,11	15,7	35,6	12,2	4,9	2,77	3,97
36,5	79,8	12,8	11,4	21,4	5,31	10,6	25,9	4,8	4,83	4,7	2,48
41,9	85,7	58,7	12,3	58,7	27,4	8,66	43,5	13,1	0,13	12,3	4,15
85,4		21,5	26		2,37	45,2		6,28	5,63		2,09
82,6		49,7	43,7		7,9	54,2		2,46	16,9		2,33
54,3		21,7	10,2		8,72	20		0,64	1,77		2,78
71,7			31,2			41,3			9,13		

Fig. 4E

Healthy IL12-p40

IL28B CC	IL28B CT	IL28B TT
83,60802	387,6784	199,8436
256,9417	52,1134	323,1031
295,0072	144,7846	419,6262
302,9375	377,0291	
365,7001	469,4738	
510,9379		

Fig. 4E

Healthy IL18

IL28B CC	IL28B CT	IL28B TT
74,4124	91,9212	39,3948
166,3336	78,7896	166,3336
96,2984	65,658	170,7108
140,0704	140,0704	21,886
30,6404	179,4652	
218,86	122,5616	
	109,43	

clinical data

AST IL28B CC	AST IL28B CT	AST IL28B TT	ALT IL28B CC	ALT IL28B CT	ALT IL28B TT	gGT IL28B CC	gGT IL28B CT	gGT IL28B TT	AP IL28B CC	AP IL28B CT	AP IL28B TT	billirubin IL28B CC	billirubin IL28B CT	billirubin IL28B TT
49	134	43	106	206	92	56	114	100	70	72	94	0,69	0,63	0,81
59	278	229	86	138	130	92	95	516	83	90	n.d.	0,67	1,04	5,86
63	28	39	53	27	38	101	134	50	93	77	62	1,18	0,42	0,47
38	44	153	47	67	240	34	93	84	58	61	96	0,8	0,65	0,58
28	42	89	39	83	149	56	257	64	116	55	96	0,26	0,68	0,38
41	76	107	118	62	146	47	73	204	96	121	90	0,45	0,59	0,4
23	86	40	19	79	54	42	179	90	49	107	81	0,94	0,52	0,96
18	96	56	27	84	56	26	152	94	64	99	163	0,47	0,84	1,31
117	38	37	172	64	60	126	57	105	73	108	64	0,44	0,81	0,43
62	175	69	56	273	112	69	291	78	59	111	79	1,07	0,71	0,36
35	24	56	47	27	81	36	53	121	56	58	108	1	0,23	0,61
88	22	112	76	41	120	158	112	154	158	42	117	1,39	0,74	0,53
22	n.d.	145	30	38	263	25	723	188	63	n.d.	91	0,69	0,41	0,43
25	34	101	31	48	69	36	20	274	63	52	192	0,71	0,49	0,63
104	80	56	45	221	57	268	99	196	109	110	97	0,88	0,52	0,76
86	27	37	151	33	55	23	53	24	62	71	201	0,49	0,26	0.98
68	9	26	146	11	49	57	311	26	89	127	35	0,58	n.d.	1,02
41	54		47	104		31	92		58	86		0,44	0,64	
39	66		41	79		31	72		55	122		0,62	0,61	
71	84		31	175		29	182		113	81		1,31	0,44	
29	21		52	34		32	32		92	84		0,37	0,24	
60	75		57	31		83	293		107	146		0,43	1,14	
19	22		34	23		58	48		86	105		0,38	0,26	
93	46		83	40		76	31		172	90		1,03	0,42	
81	27		119	39		67	393		84	75		0,85	0,3	
52	28		126	18		34	195		45	50		0,42	0,38	
215	158		355	168		145	62		56	109		0,86	0,72	
178	39		367	42		314	183		84	127		0,73	0,35	
	24			37						72			0,36	
	34			44						63			0,4	

HCV load (IU/ml) IL28B CC	HCV load (IU/ml) IL28B CT	HCV load (IU/ml) IL28B TT	HCV Genotype IL28B CC	HCV Genotype IL28B CT	HCV Genotype IL28B TT	Age IL28B CC	Age IL28B CT	Age IL28B TT	Sex (m=male; w= female) IL28B CC	Sex (m=male; w= female) IL28B CT	Sex (m=male; w= female) IL28B TT
4976334	608698	3531277	1a	1a	1a/4a	36	57	53	m	m	m
2734995	266073	228042	1b	2a	1a	61	56	64	m	m	w
11369077	814117	294947	1a	2a	1b/4a	55	79	50	m	m	w
2615515	2245188	736700	1b	1a	1b	49	52	58	m	m	w
5218106	4335288	14	1a	4	1b	67	46	60	w	w	m
4316178	403384	723834	1a	1b	1a	51	67	56	m	w	m
457008	180424	425086	1b	1b	1b	73	39	53	m	m	m
3029	5791994	300861	1a	1b	1a	46	71	35	m	m	w
5320617	879877	1647906	1a	1a	1b	69	50	54	m	m	m
1926823	191132	15068	1b	1a	1a	45	55	60	w	m	m
2051041	233573	1102485	1b	1b	1b	37	63	53	w	m	w
6310	1041833	825894	1a	1	1b	32	37	45	m	m	w
1687	74424	169750	1b	1a	1b	49	55	42	m	m	m
2035383	1195731	2741293	2a	1a	1b	70	59	25	w	w	m
829704	4652	2232754	1b	1b	4h	75	53	58	m	m	m
1807391	5967	4804948	1b	2	1b	81	55	74	w	m	m
565336	384615	31995	2	1a	1b	63	59	45	m	m	w
1096	4234395		1b	1a		56	78		w	m	
2289762	1726338		1b	2		79	60		m	w	
266414	1039116		1a	1a		76	62		w	m	
5555964	768740		1a	1		48	55		w	w	
1430836	1744		1a	1b		56	55		w	m	
2098139	8256		1b	1b		57	67		w	w	
111472	1468242		2a	1b		56	62		m	w	
1060778	923869		1b	1b		54	63		m	w	
6263118	5664655		2a	2a		44	53		m	m	
1869214	346945		1b	1b		43	56		m	m	
7620549	5468128		1a	4a		38	77		m	w	
	516976			1a			61			m	
	2479350			1a			58			m	
